# Cyberpharmacies and the role of the US Food And Drug Administration

**DOI:** 10.2196/jmir.3.1.e3

**Published:** 2001-01-31

**Authors:** Jane E Henney

**Affiliations:** ^1^Commissioner of Food and DrugsFood And Drug AdministrationUSA

**Keywords:** Internet, Drug and Narcotic Control, Prescriptions, Drug, Commerce, Physician's Practice Patterns, Impotence, United States Food and Drug Administration, Phosphodiesterase Inhibitors, Piperazines, Quality of Health Care

## Abstract

The sale of consumer products over the Internet has grown rapidly, including the sale of drugs. While the growth in online drug sales by reputable pharmacies is a trend that may provide benefits to consumers, online drug sales also present risks to purchasers and some unique challenges to regulators, law enforcement officials and policy makers. The Food and Drug Administration (FDA or the Agency) is concerned about the public health implications of Internet drug sales, and we are responding to these concerns as part of our overall goal of developing and implementing risk-based strategies to protect public health and safety.

Although other products regulated by the Agency, such as medical devices, medical test products, foods, dietary supplements and animal drugs also are sold online, this paper focuses on online drug sales. We discuss the advantages and risks of online drug sales, outline FDA's authority and enforcement activities in this area, and describe new initiatives we are taking to better respond to the regulatory challenges we face.

## Benefits of Online Drug Sales

The use of the Internet by our nation's citizens, from school age children to seniors, has opened up vast new opportunities for the exchange of information and for enhancing commerce in all types of consumer products. Electronic mail and chat groups have dramatically facilitated communications. Information gathering that once took hours or days of research, whether for a student's homework assignment or to look up information on the medical condition of a family member, can now be accomplished in minutes.

The Internet is rapidly transforming the way we live, work, and shop in all sectors of the economy. In the health sector, tele-medicine allows people in remote areas to access the expertise of doctors in the nation's finest academic health centers. The Internet permits an increasing number of individuals to obtain a plethora of medical information that often helps them to understand health issues and treatment options. In fact, more than 22 million Americans used the Internet last year to find medical information, either in documentary resources or through online discussions with health professionals. According to Investor's Business Daily, 43 percent of web surfers access health care data online each year. Conducting research regarding their health concerns is the sixth most common reason that people use the Internet, and according to the market research firm, Cyber Dialogue Inc., this number is growing by 70 percent a year.

The increasing recognition of the Internet as a legitimate and important vehicle for drug sales is evidenced by the recent activity of major drugstore companies and Internet retailers in financing, supporting, and sponsoring online pharmaceutical outlets. Last year, for example, CVS Corporation acquired the online pharmaceutical retailer Soma.com and merged the online retail sites of the two companies. We expect this expansion of the online drug sales industry to continue.

Prescription drug sales on the Internet can provide tremendous benefits to consumers. These benefits are many and include: access to drugs for the disabled or otherwise home-bound, for whom a trip to the pharmacy can be difficult; the convenience of shopping 24 hours a day; an almost unlimited number of products for customers; and privacy for those who don't want to discuss their medical condition in a public place. Hyperlinks and search programs provide online customers with written product information and references to other sources of information much more easily than the traditional storefront. Finally, as the use of computer technology to transmit prescriptions from doctors to pharmacies expands, a reduction in prescription errors may be possible.

While online pharmaceutical sales will be important for some customers, it must be noted that the traditional "brick and mortar" pharmacy offers benefits or services that are often not available through the Internet, such as immediate access to prescription drugs needed for immediate treatment. These pharmacies will undoubtedly remain an essential component in the delivery of effective health care.

The challenge for government at both the state and federal level is to pursue policies that will allow legitimate electronic commerce to flourish but provide that safety is assured. Consumers will have confidence in the quality of the medical prescription and in the medicine delivered because the protection for online consumers is equivalent to the safeguards of the traditional local pharmacy and the practice of medicine and pharmacy.

## Concerns About Online Sales

As beneficial as this new technology can be, the Internet also creates a new marketplace for activity that is already illegal, such as the sale of unapproved new drugs, prescription drugs dispensed without a valid prescription, and products marketed with fraudulent health claims. As FDA considers the issues related to online drug sales, we recognize that there are various types of these Web sites. Many sites focus on selling prescription drugs and have been referred to by some as "Internet pharmacies." These sites offer for sale either FDA-approved prescription drug products, or in some cases, unapproved, illegal versions of prescription drugs. While the sales sites of legitimate, properly licensed pharmacies provide benefits to consumers, those that are unlicensed or otherwise engaged in the illegal dispensing of prescription drugs pose a serious threat to the health and safety of American citizens. Other drug sales sites do not sell prescription drugs, but may offer for sale unapproved drug products, products making fraudulent health claims, or drugs for recreational use. Examples of these sites are those that sell products containing gamma hydroxy butyrate (GHB), an unapproved drug used recreationally, for body building and for incapacitating the victims of sexual assaults, or sites that offer unproven cancer therapies. It should be noted that with regard to GHB, Congress recently passed legislation which the President signed in February to place GHB in Schedule 1 of the Controlled Substances Act. While the increase in "Internet pharmacy" sites engaged in illegal sales is seen by some as a particularly potent threat, FDA considers the non-pharmacy sites to be just as harmful, or in some cases more so, and we have moved aggressively against them.

The unique qualities of the Internet, including its broad reach, relative anonymity, and ease of creating new Web sites or removing old ones, pose new challenges for the enforcement of existing laws. FDA has found that most drug sales websites are actually made up of multiple related sites and links, thereby making investigations much more complex and resource intensive. The global nature of the Internet creates particular problems for effective law enforcement. Different approaches to drug approval and marketing in foreign countries further complicate law enforcement issues for U.S. officials. FDA and other U.S. government agencies need to work closely with foreign governments to share information and to develop mechanisms for cooperative law enforcement.

### FDA Authority

The establishment of FDA as it exists today grew out of a time early in the century when consumers were victimized by dishonest purveyors of fraudulent potions and compounds that were ineffective, dangerous, or both. A system of drug regulation was established in the United States that has served us well. Under this system, FDA reviews new drugs to assess their safety and efficacy. In addition, certain types of drugs must be prescribed and dispensed by only licensed health care professionals. The prescribing requirement is based on the principle that certain drugs have risks of such significance associated with them that they should be administered only under the supervision and recommendation of a "learned intermediary" - that is, a licensed practitioner with the education and training necessary to oversee the administration of potentially harmful drug products. Similarly, these products may only be dispensed by a licensed professional that can help to assure proper dosing and administration, and can provide important information on the drug's use to patients. These requirements are crucial components of the risk management system for drugs in the United States. The types of unlawful conduct involving online drug sales that FDA has identified are similar to unlawful activities that occur in other sales contexts. Under the Federal Food, Drug, and Cosmetic Act, FDA has the legal authority to take action against:

- the importation, sale, or distribution of an adulterated or misbranded drug;
- the importation, sale, or distribution of an unapproved new drug;
- illegal promotion of a drug;
- the sale or dispensing of a prescription drug without a valid prescription; and,
- counterfeit drugs.

When the Internet is used for an illegal sale, FDA, working with the Department of Justice, must establish the same elements of a case, develop the same charges, and take the same actions as it would if another medium, such as a storefront or a magazine, had been used. FDA has investigated and referred cases for criminal prosecution and initiated civil enforcement actions against online sellers of drugs and other FDA-regulated products, particularly sellers of drugs not approved by the Agency. As will be described later, FDA has significantly expanded its enforcement activities during this past year with regard to online drug sales.

### State Regulation of Practice of Medicine, Pharmacy and Dispensing of Drugs

Similarly, the States have enacted laws regulating the practice of pharmacy and the practice of medicine in order to protect patients from harm resulting from the use of unsafe drugs, counterfeit drugs, and the improper practice of medicine and pharmacy. Under many of these laws, to receive a prescription drug for the first time, a patient generally must be physically examined by a licensed health care practitioner who determines the appropriate treatment and issues a prescription for an FDA-approved drug. The patient then has the prescription filled by a registered pharmacist working in a licensed pharmacy that meets state practice standards.

### Use of the Internet to Bypass the Regulatory System

Even with these federal and state systems in place, there are those who try to circumvent established safeguards, and the Internet provides them with new opportunities for doing so. It is fair to say that the speed and ease of ordering products on the Internet that attracts consumers can likewise entice unscrupulous sellers to use the Internet as their new medium of choice. Individuals not licensed to sell prescription drugs can easily create Web sites that appear to represent legitimate pharmacies. The fact that operators can easily change the location and appearance of their Internet sites makes enforcement all the more difficult. Unlike other forms of electronic commerce, the unauthorized sale of prescription and unapproved drugs poses a potential threat to the health and safety of consumers.

Patients who buy prescription drugs from an illegitimate site are at risk of suffering adverse events, some of which can be life-threatening. These risks include potential side effects from inappropriately prescribed medications, dangerous drug interactions or contaminated drugs, as well as the possible ill effects of impure or unknown ingredients found in drugs manufactured under substandard conditions. Further risk to patients is posed by their inability to know what they are really getting when they buy some of these drugs. Although some patients may be purchasing genuine product, some may unknowingly be buying counterfeit copies that contain inert ingredients, outdated legitimate drugs that have been diverted to illegitimate resellers, or dangerous sub-potent or super-potent versions that were improperly manufactured. Moreover, consumers who are desperate for a cure to a serious medical problem may be more susceptible to purchasing an unapproved product.

FDA is concerned about the proliferation of sites that substitute a simple online questionnaire for a face-to-face examination and patient supervision by a health care practitioner. According to the American Medical Association, health care practitioners who offer a prescription for a patient they have never seen before, based solely on an online questionnaire, generally do not meet the appropriate medical standard of care. Additionally, the use of such questionnaires may jeopardize the availability of legal protections for privacy of medical records.

The Agency is equally concerned that in some Internet transactions, there is an apparent absence of any health professional/patient relationship. This is a particular concern where the prescription involves a first-time use by a patient or where the patient may be taking other medications. FDA believes that the selection of prescription drug products or treatment regimens for a particular patient should be made with the advice of a licensed health care practitioner familiar with the patient's current health status and past medical history. In situations where a customary physician-patient relationship does not exist, the patient is essentially practicing self-diagnosis. Consequently, the risk of negative outcomes such as harmful drug interactions, contraindications, allergic reactions or improper dosing is greatly magnified.

### Jurisdictional Issues

Internet technology can obscure the source of the product as well provide some degree of anonymity to persons responsible for making and shipping the product. The participants in a transaction can be widely dispersed geographically (in different States or countries) and they may never meet. If one or more participants in the transaction are located outside of the United States, the task of regulating the activity is further complicated.

The sale of drugs to U.S. residents via foreign Web sites is an extremely challenging area. Some medications sold on the Internet may be legal in foreign countries but not approved for use in the United States, and some products may include addictive and dangerous substances. Products not approved for sale in the United States often do not conform to the good manufacturing practice and quality assurance procedures required by U.S. laws and regulations, and it is illegal for a foreign pharmacy to ship such drugs into the United States. Foreign sales pose the most difficult challenge for U.S. law enforcement because the seller is not within U.S. boundaries.

FDA efforts are mostly limited to requesting the foreign government to take action against the seller of the product, or asking the Customs Service to stop the imported drug at a U.S. port-of-entry.

Other governments are also struggling with how to address the problem of illegal drug sales over the Internet. For instance, pharmaceutical industry officials in Italy are recommending that the issue be addressed by the European Community as a whole.

Last month, the New Zealand Health Ministry began to look at options to prevent pharmaceuticals from being dispensed from New Zealand to overseas consumers without a prescription, after a court decision revealed a loophole that prevents regulators from preventing the practice.

## FDA's Internet Drug Sales Action Plan

In July 1999, FDA adopted, and has since been implementing, an Internet Drug Sales Action Plan to expand and improve the activities of the Agency in addressing the unlawful sale of drugs over the Internet. This plan is based on internal deliberations, meetings with federal and state regulatory and law enforcement bodies, as well as organizations representing consumers, health care practitioners, and the pharmaceutical and pharmacy industries. Details of the action plan's elements and FDA's activities in implementing them are as follows.

### Engage in Public Outreach

Consumers buy drugs on the Internet for different reasons, and some may be targets of unscrupulous business practices, such as the selling of unsafe, unapproved, expired, counterfeit, or otherwise illegal drugs. Public outreach offers one mechanism by which the Agency can help protect consumers from dangerous or inappropriate drugs. In the year 2000, FDA has launched a new media campaign about safe ways to purchase pharmaceutical products over the Internet. The campaign includes placing advertisements on health related Web sites; taping public service announcements for distribution to television stations nationwide; and developing a "safety checklist" to be posted online and distributed through health care providers and consumer advocacy organizations.

### Engage in Professional Outreach and Partnering

The National Association of Boards of Pharmacy (NABP) has implemented a new program to verify the legitimacy of Internet sites dispensing prescription drugs. The program, known as the Verified Internet Pharmacy Practice Sites, or VIPPS, provides a NABP "seal of approval" to sites meeting state licensure requirements and NABP's standards. Over time, this seal of approval may help to assure consumers that the designated sites are offering FDA approved pharmaceuticals. The VIPPS program is voluntary.

### Cooperate Internationally

Because FDA and the other federal agencies possess limited investigatory jurisdiction over sellers in foreign countries, they must work with foreign governments to bring action against such individuals. Internet crime and the practice of online pharmacy are a growing concern throughout the international law enforcement community. FDA's Office of Criminal Investigations (OCI) maintains ongoing liaison with numerous government agencies in Canada, the United Kingdom, Spain, Germany, Belgium, the Netherlands, Ireland, Brazil, Singapore, and others.

An example of this cooperation involved OCI contact with authorities in a Pacific Rim country where a Web site operator alleged that he used the services of two legitimate doctors to review his online questionnaire. Through our foreign counterparts, we were able to have the doctors interviewed. Both denied any involvement in the scheme, thus exposing the operator to possible mail and wire fraud or other charges.

In another case, OCI made an undercover purchase of drugs from a site operating out of a European country. The site made no pretense of a medical review. OCI was looking for a domestic connection for charges in the United States. While none was found, our contacts with the health authorities in that country resulted in their initiation of a criminal investigation.

Finally, OCI is involved in two cases with U.S. Customs Service overseas offices regarding foreign Web sites selling prescription and controlled pharmaceuticals. Enforcement activity by Customs resulted in numerous arrests and the seizure of over 1.5 million pills and several computers.

### Customize and Expand Enforcement Activity

FDA's emerging role in regulating online drug sales is consistent with its traditional regulatory role. Existing approaches to enforcement, including close cooperation with state agencies, are being adapted to focus more effectively on the problems posed by online drug sales. An effective Internet enforcement process requires establishing priorities, identifying and monitoring potentially violative Web sites, and making appropriate referrals for criminal prosecution and/or civil enforcement actions. FDA is enhancing its enforcement efforts by - amongst others - undertaking the following actions.

*Establishing Priorities*

FDA has initially focused its online drug sales-related enforcement activities to the following areas, particularly where there is a significant public health risk:

- Unapproved new drugs,
- Health fraud, and
- Prescription drugs sold without a valid prescription.

*Improving Data Acquisition*

FDA has increased its capability to monitor the Internet and identify potentially violative sites through the use of various search tools and by upgrading its data handling capabilities. This is helping the Agency to better understand the kind and extent of unlawful conduct on the Internet and to more accurately assess whether its enforcement efforts have had an impact on illegal Internet behavior.

In an attempt to better comprehend the universe of Web sites selling drugs, the Office of Criminal Investigations reviewed thousands of Web sites early this year and identified approximately 326 Web sites involved in the sale of drug products. This review was based on a search of Web sites performed by Internet search software, which was followed by a manual review of sites that appeared to involve the sale of drug products. Because new Web sites are put up everyday and old ones are taken down, the total number of these sites is subject to change and will not be consistent over time. Additionally, because OCI's technology and methodology probably differs from that used in studies by other organizations, the results of this study are not directly comparable to other studies.

*Coordinating Case Assessment*

In June 1999, FDA established a case assessment, or "triage" team with representatives from the Office of Enforcement and OCI within the Office of Regulatory Affairs (ORA), the Center for Drug Evaluation and Research (CDER), the Office of the Chief Counsel (OCC), and the Office of Policy. Under the triage process, FDA obtains leads on potentially violative sites from internal Internet monitoring activity, state, other federal or foreign law enforcement agencies, consumers, Congress, and the press. The triage team evaluates the leads and decides whether they should initially be pursued through a civil or criminal investigation. Priority is given to cases involving unapproved new drugs, health fraud, prescription drugs sold without a valid prescription, and products with the potential for causing serious or life-threatening reactions. The triage team makes referrals, when appropriate, to FDA's civil and criminal enforcement units for follow-up.

The triage process results in a better coordination of criminal and civil enforcement actions at the appropriate Agency components and reduces overlapping effort. This process better ensures that decisions are made in a timely way, with an appropriate balance in terms of achieving a maximum deterrent effect while taking action, if needed, to remove harmful products from the market. The team will continue to oversee Internet-related enforcement activities while they are being investigated and will ensure that they are brought to appropriate completion. In addition, the scope of this group is being broadened to include all FDA-regulated products.

### Results to Date

Using information generated by Internet searches, as well as leads from all parts of the Agency, other state and federal law enforcement units, and the public, FDA has performed at least cursory reviews of thousands of Web sites related to drug sales. FDA has evaluated for possible regulatory or criminal action well over 400 sites and has taken enforcement action on many of those sites, as follows.

Civil or regulatory enforcement actions are pursued by ORA and CDER's Office of Compliance in cooperation with OCC. Currently, ORA and the Office of Compliance have at least 40 sites under active review for possible regulatory or civil action. Regulatory action has been taken on 49 sites as follows. Twenty-three warning letters have been sent by the Office of Compliance to domestic online sellers. A warning letter is a written communication from FDA notifying an individual or firm that the Agency considers one or more products, practices, processes, or other activities to be in violation of the FD&C Act, or other relevant statutes, and that failure of the responsible party to take appropriate and prompt action to correct and prevent any future repeat of the violation may result in administrative and/or regulatory enforcement action without further notice.

Additionally, the Office of Compliance has sent 13 "cyber letters" to operators of foreign-based Internet sites offering to sell online prescription drugs. These sites may be engaged in illegal activity such as offering to sell prescription drugs to U.S. citizens without valid (or in some cases without any) prescriptions. Cyber letters, which are sent over the Internet to the suspect Web sites, warn these operators that they may be engaged in illegal activities, and informs them of the laws that govern prescription drug sales in the United States. Hard copies of each "cyber" letter are sent to the Web site operator, the U.S. Customs Service, and to regulatory officials in the country in which the operator is based. FDA already has received two responses from "cyber" letter recipients indicating that they will cease illegal activities. A third recipient has stated that it has ceased activities regarding Viagra, but it is still evaluating how it will handle other products.

Other civil and regulatory actions include the following. In cooperation with the Department of Justice (DOJ), an injunction has been imposed on the sale of a product marketed as a weight-loss aid that contains a potent thyroid hormone, which could cause heart attacks or strokes. FDA and DOJ are pursuing injunctions against the sale of other unapproved new drugs over the Internet, and unapproved cancer therapies. Additionally, eight product seizures, six product recalls, and the voluntary destruction of seven violative products have been achieved, generally pertaining to unapproved new drug products including gamma hydroxy butyrate (GHB), gamma butyrolactone (GBL), Triax, 1,4 butanediol, and laetrile. Fourteen import alerts have been issued targeting products offered by foreign online drug sellers.

The Office of Criminal Investigations, working with OCC, is responsible for investigations of pharmacy sites and other Internet drug sites whose operations involve potential criminal activity. The information collected by OCI headquarters is analyzed by the Investigative Analysis Branch. After the suspect sites are researched they are sent to the OCI field offices for investigative work, which often includes undercover buys. Further investigation determines the bona fides of the pharmacy and doctor(s), and looks at the relationship between the patient and doctor, and the doctor and pharmacy. OCI has ongoing cooperative relationships with the USCS, DEA, FBI, the Postal Inspection Service, and appropriate state law enforcement and regulatory agencies, and this has enhanced their investigative capabilities with regard to Internet drug sales.

Currently, OCI has 134 Internet related investigations, including 88 open criminal investigations and 46 preliminary investigations. Of these 134 investigations, 54 cases are investigations of sites selling prescription drugs, while 80 cases are related to various types of health fraud, or unapproved drug products such as GHB or other illegal drug sales. Thirty-six arrests and 17 convictions have resulted from OCI investigations involving products being sold over the Internet.

### Legislative Proposal

The Administration is currently completing the development of legislation to implement its Internet drug sales initiative. The underlying basis for the proposal is that online pharmacies should be licensed and operated under the same regulatory system that Congress and the States have put in place for traditional "brick and mortar" pharmacies. A key element of the initiative, therefore, is a requirement that online pharmacies post information on their Web sites about their ownership, state licensure, name of the pharmacist in charge, and a phone number where consumers can contact the pharmacist. This information mirrors what is available to the consumer at the corner pharmacy, where the license is on display and the pharmacist is available for consultation. Online pharmacies operating without the licensure required by States would be subject to both state and federal penalties.

Under this proposal, the traditional role of the States in regulating the practice of medicine and pharmacy would be maintained. It should be noted that the plan would establish only minimal new federal requirements that would not appreciably impact the day-to-day operations of legitimate pharmacies doing business online.

FDA is continuing to work with the Administration on putting this initiative into legislative language, and we look forward to working with you when legislation is forwarded to Congress.

## Conclusions

Online shopping for pharmaceutical products clearly provides certain benefits for consumers, but it also has a number of significant risks. Additionally, the nature of Internet technology presents law enforcement and policy makers with unique challenges. FDA is grappling with the challenges posed by online drug sales and with our need to carefully balance consumer access to information and products with protecting the public health. We are adapting our compliance and enforcement techniques to the new electronic marketplace and we will continue to evaluate what changes in our procedures, regulations, or the law might be appropriate. We want to ensure, as much as possible, that the protections afforded to consumers who purchase drugs from their corner drugstore are extended to consumers in the electronic marketplace.

